# Radiologic Assessment of Acute Aortic Syndrome With Multiple Penetrating Atheromatous Ulcers: A Case Report

**DOI:** 10.7759/cureus.77633

**Published:** 2025-01-18

**Authors:** Harminder Sandhu, Derrick L Barr, Lauri Tyre

**Affiliations:** 1 Radiology, Michigan State University College of Osteopathic Medicine, Detroit, USA; 2 Diagnostic Radiology, Trinity Health, Pontiac, USA; 3 Diagnostic Radiology, Wayne State University School of Medicine, Detroit, USA

**Keywords:** acute aortic syndrome, cardio vascular disease, cardiovascular radiology, general radiology, multiple penetrating atheromatous ulcers, penetrating aortic ulcer, penetrating atheromatous ulcer, radiology case report, radiology finding, radiology research

## Abstract

Acute aortic syndromes (AAS) include life-threatening conditions like penetrating atheromatous ulcer (PAU), which occurs when an atherosclerotic plaque erodes through the aortic wall. This can lead to complications such as intramural hematoma, pseudoaneurysm, or aortic rupture, especially in the ascending aorta. PAUs typically affect older males with atherosclerosis and are most commonly found in the lower descending thoracic aorta, with multiple PAUs being rare. This report highlights a case involving the incidental discovery of multiple PAUs and an associated ductus diverticulum, and it discusses their presentation and management. An 88-year-old male with a history of hypertension, diabetes, and pulmonary fibrosis presented with right-sided upper quadrant and chest pain radiating to the back. Initial imaging suggested progression of pulmonary fibrosis or an infection, but a subsequent CT angiogram revealed at least 12 focal outpouchings in the distal aortic arch and proximal descending thoracic aorta, compatible with PAUs. Additionally, a ductus diverticulum was identified. The patient was managed with high-dose statin therapy and scheduled for follow-up CTA in three months. This case highlights the incidental discovery of multiple PAUs and a ductus diverticulum, underscoring the rarity and complexity of PAU presentations. It emphasizes the importance of including PAU in the differential diagnosis for patients presenting with chest pain, especially when symptoms are atypical or overlap with other conditions. Early identification and management are crucial to prevent severe complications such as aortic dissection or rupture.

## Introduction

Acute aortic syndromes (AAS) include three life-threatening conditions involving the aorta with similar characteristics including, acute aortic dissection, intramural hematoma (IMH), and penetrating aortic or atheromatous ulcer (PAU) [1]. These conditions are caused by a common pathway leading to the breakdown of the internal elastic lamina and media of the aorta [1,2]. PAU occurs due to the ulceration of an aortic atherosclerotic plaque, which then penetrates the internal elastic lamina and media of the vessel [1]. PAUs are uncommon manifestations of AAS, representing 2-7% of all AAS cases, and they can lead to IMH, pseudoaneurysm, and aortic rupture through the progressive enlargement of the aorta, with an increased risk in the ascending aorta (Type A PAU) [1,2]. This condition usually affects older male patients with a history of tobacco smoking, hypertension, coronary artery disease, and chronic obstructive pulmonary disease [1,3]. PAU usually presents in the setting of extensive and widespread atherosclerosis with the most common location being in the middle and lower descending thoracic aorta (Type B PAU) [1,3]. PAUs are infrequently found in the arch of the aorta or abdominal aorta, and rarely in the ascending aorta [1,2]. Multiple PAUs are infrequently identified simultaneously, with rarity increasing with the number of PAUs [3-10]. This report presents a case of the incidental discovery of multiple PAUs with an associated ductus diverticulum and discusses the presentation and management of this AAS.

## Case presentation

The patient is an 88-year-old male with a past medical history of hypertension, diabetes mellitus type 2, hyperlipidemia, hypothyroidism, pulmonary embolism, and pulmonary fibrosis, who is on as-needed oxygen. He initially presented to the emergency room with right-sided upper quadrant pain and chest pain radiating to the back. The pain worsened with deep breathing. The patient was tachypneic but otherwise hemodynamically stable. There was no elevation in troponin levels, and no ischemic ECG changes were noted; the ECG demonstrated normal sinus rhythm. Labs were remarkable for hyponatremia, hypomagnesemia, and mild leukocytosis, but procalcitonin was negative (Table 1).

**Table 1:** Relevant laboratory results with corresponding reference ranges

Lab (units)	Result	Reference range
Sodium (mmol/L)	132	136-145
Magnesium (mg/dL)	1.5	1.7-2.2
White blood cells (WBCs) (K/mcL)	11.9	4.0-11.0
High-sensitivity troponin I (ng/L)	6	<19 (females) / <34 (males)
Procalcitonin (ng/mL)	0.03	<0.05

**Figure 1:**
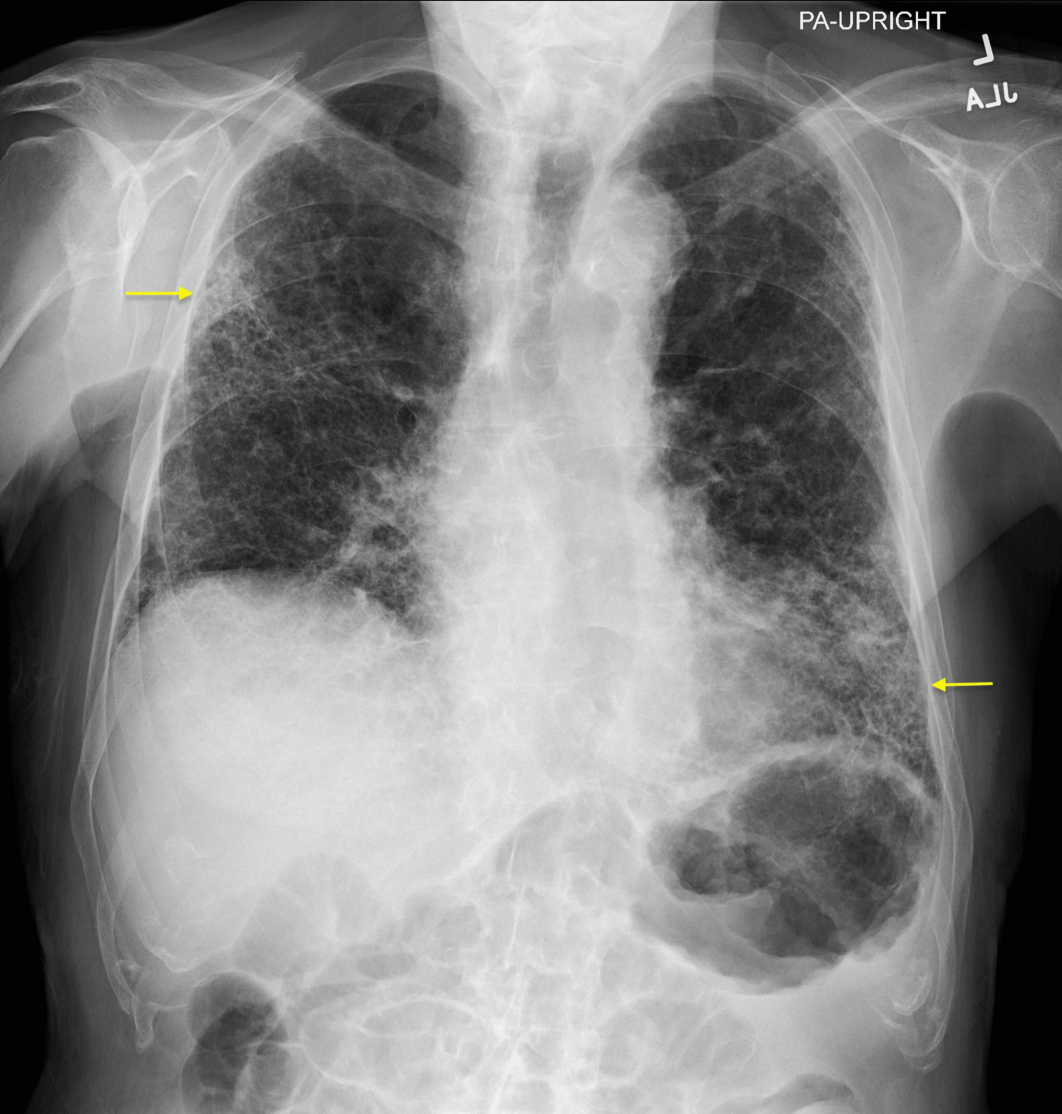
Chest X-ray (posteroanterior view) showing patchy interstitial opacities in the bilateral lung fields, with increased prominence in the right lateral upper lobe and left lower lobe (yellow arrows)

**Figure 2:**
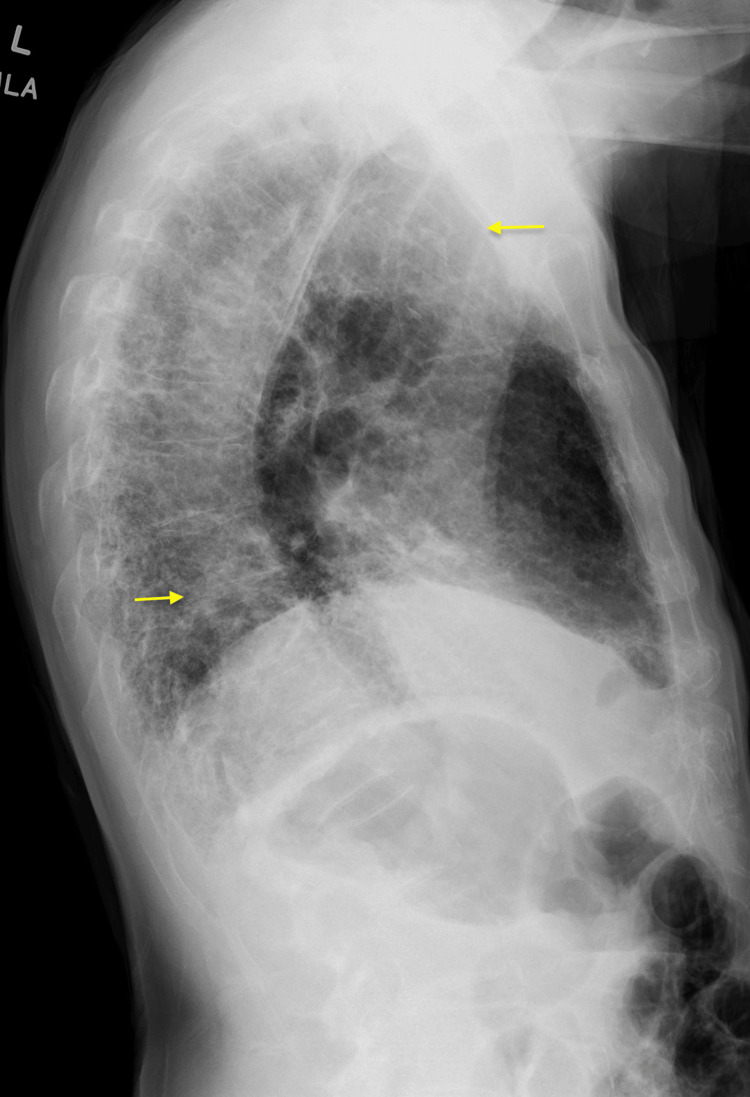
Chest X-ray (lateral view) showing patchy interstitial opacities in the bilateral lung fields, with increased prominence in the right lateral upper lobe and left lower lobe (yellow arrows)

**Figure 3:**
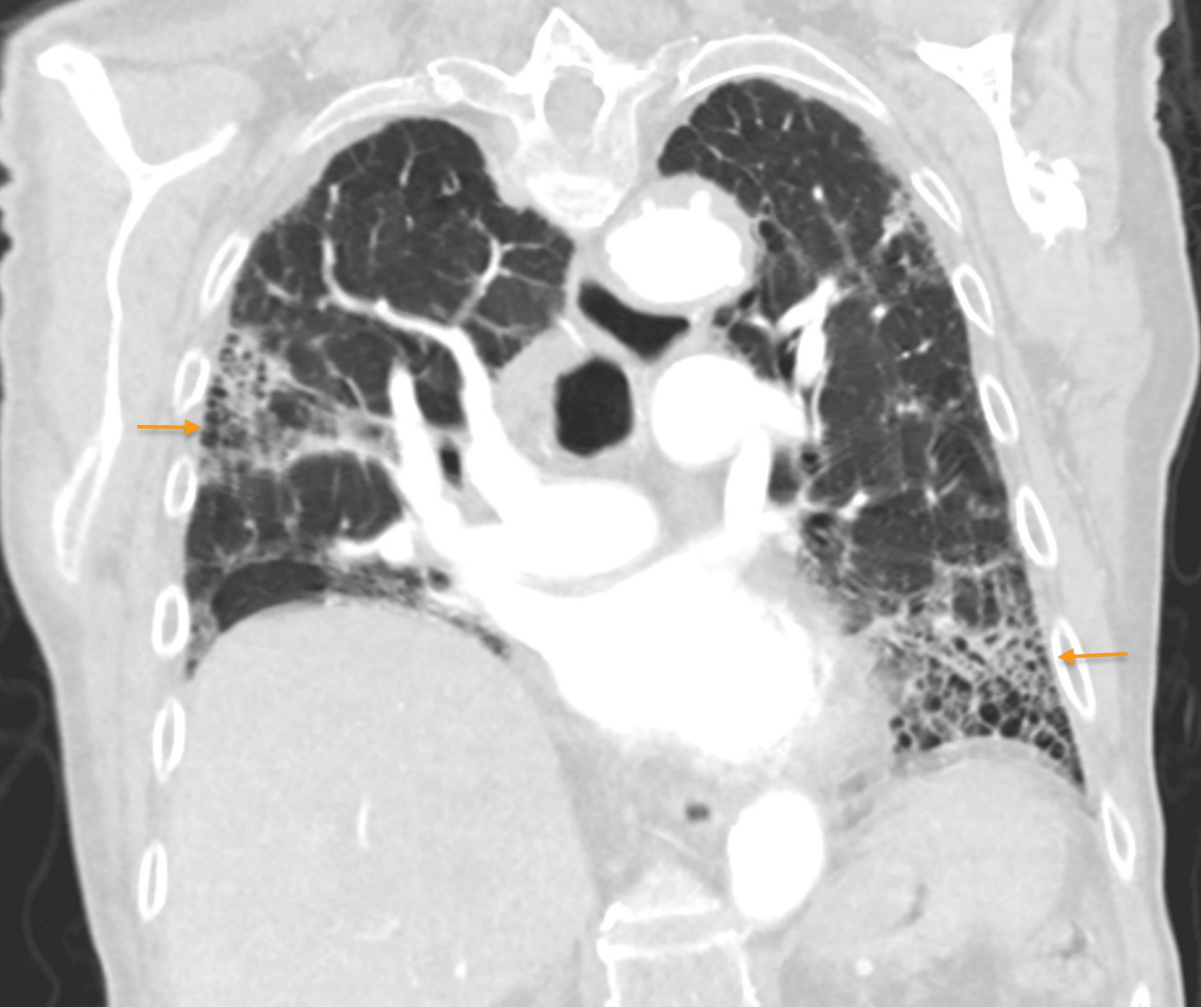
CTA of the chest with contrast (coronal view) demonstrating the usual interstitial pneumonia pattern of pulmonary fibrosis, most pronounced in the right lateral upper lobe and left lower lobe (orange arrows) CTA: computed tomography angiogram

**Figure 4:**
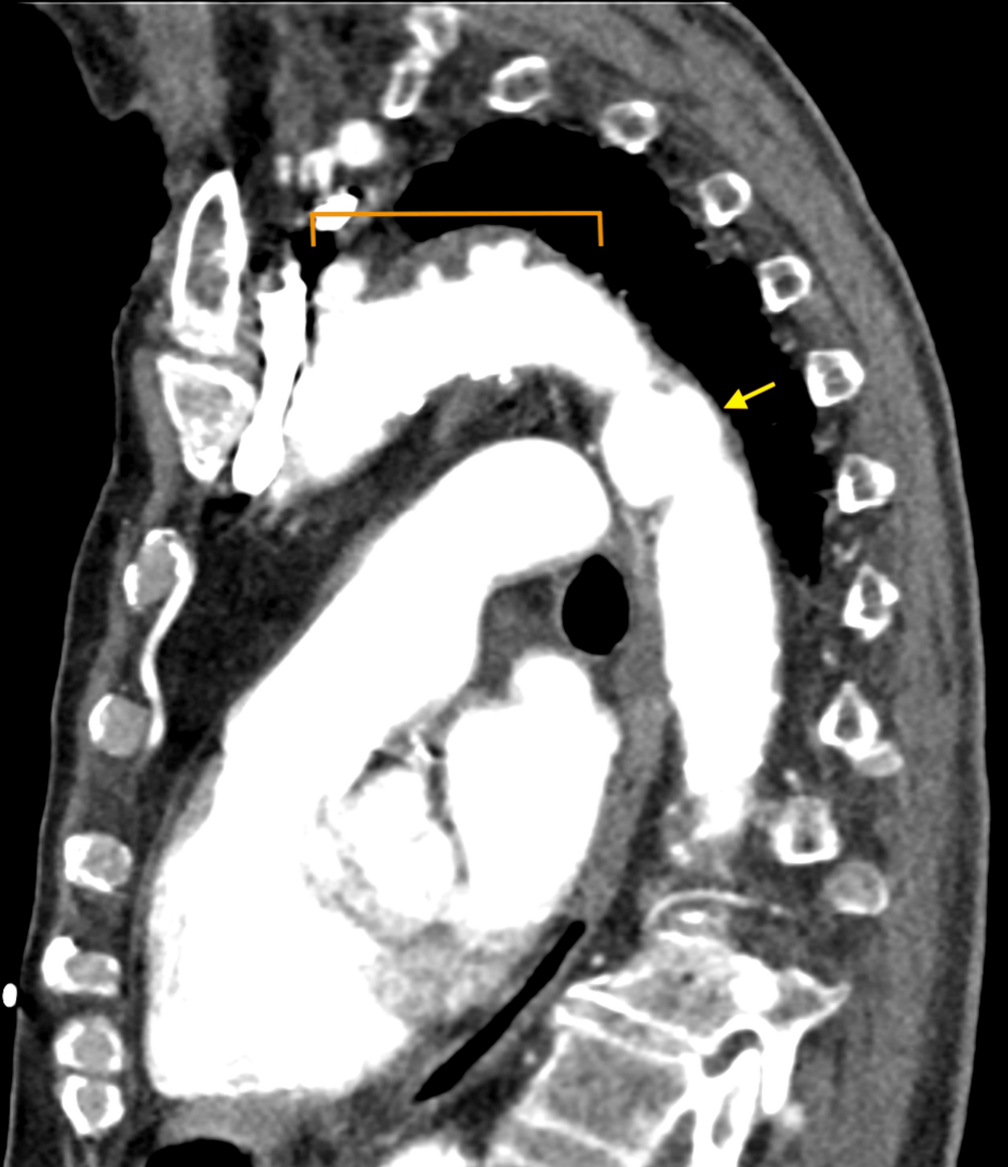
CTA of the chest with contrast (sagittal view) showing multiple focal outpouchings from the distal aortic arch and proximal descending thoracic aorta (orange bracket), as well as a ductus diverticulum in the proximal descending thoracic aorta (yellow arrow) CTA: computed tomography angiogram

**Figure 5:**
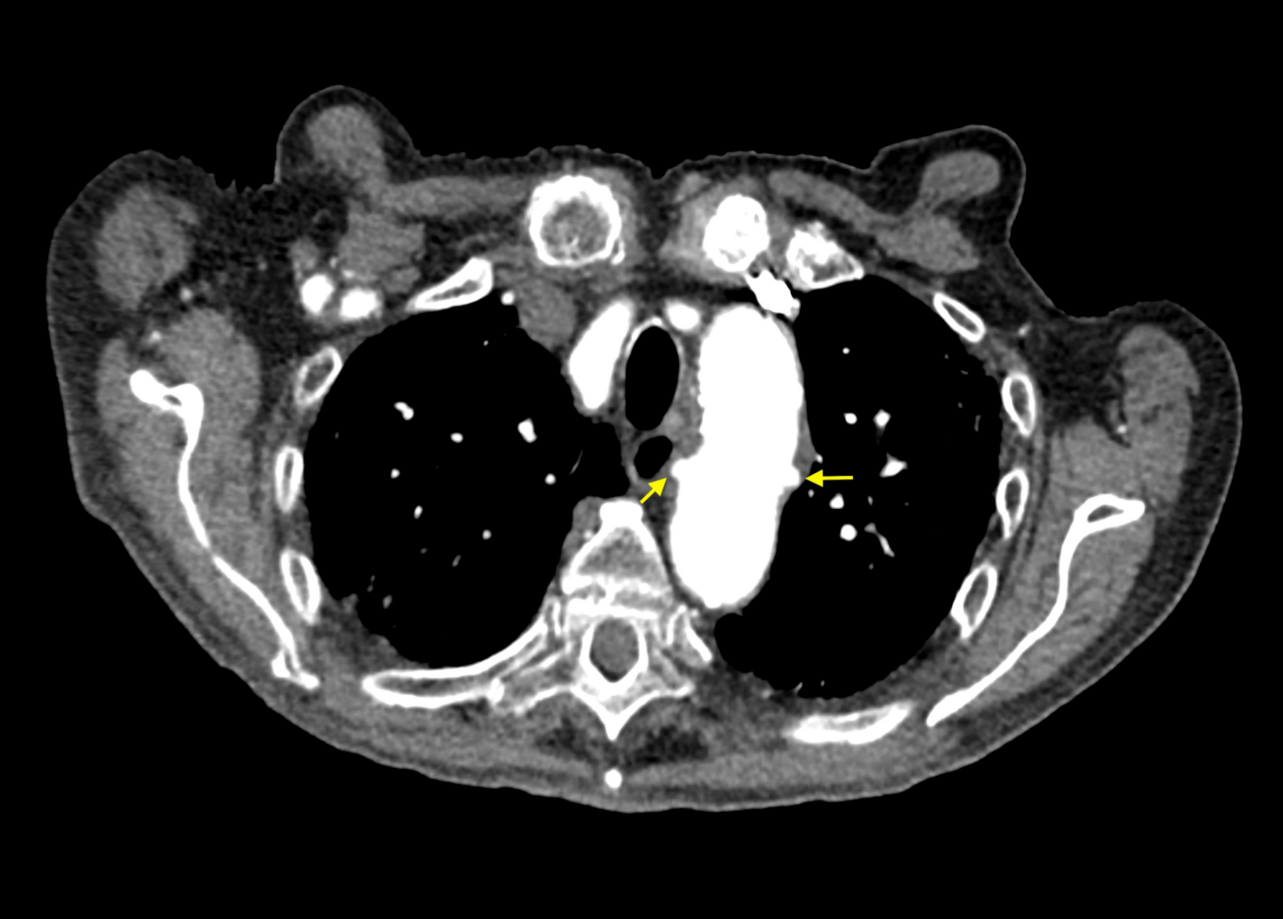
CTA of the chest with contrast (axial view) showing multiple focal outpouchings in the distal aortic arch and proximal descending thoracic aorta (yellow arrows) CTA: computed tomography angiogram

**Figure 6:**
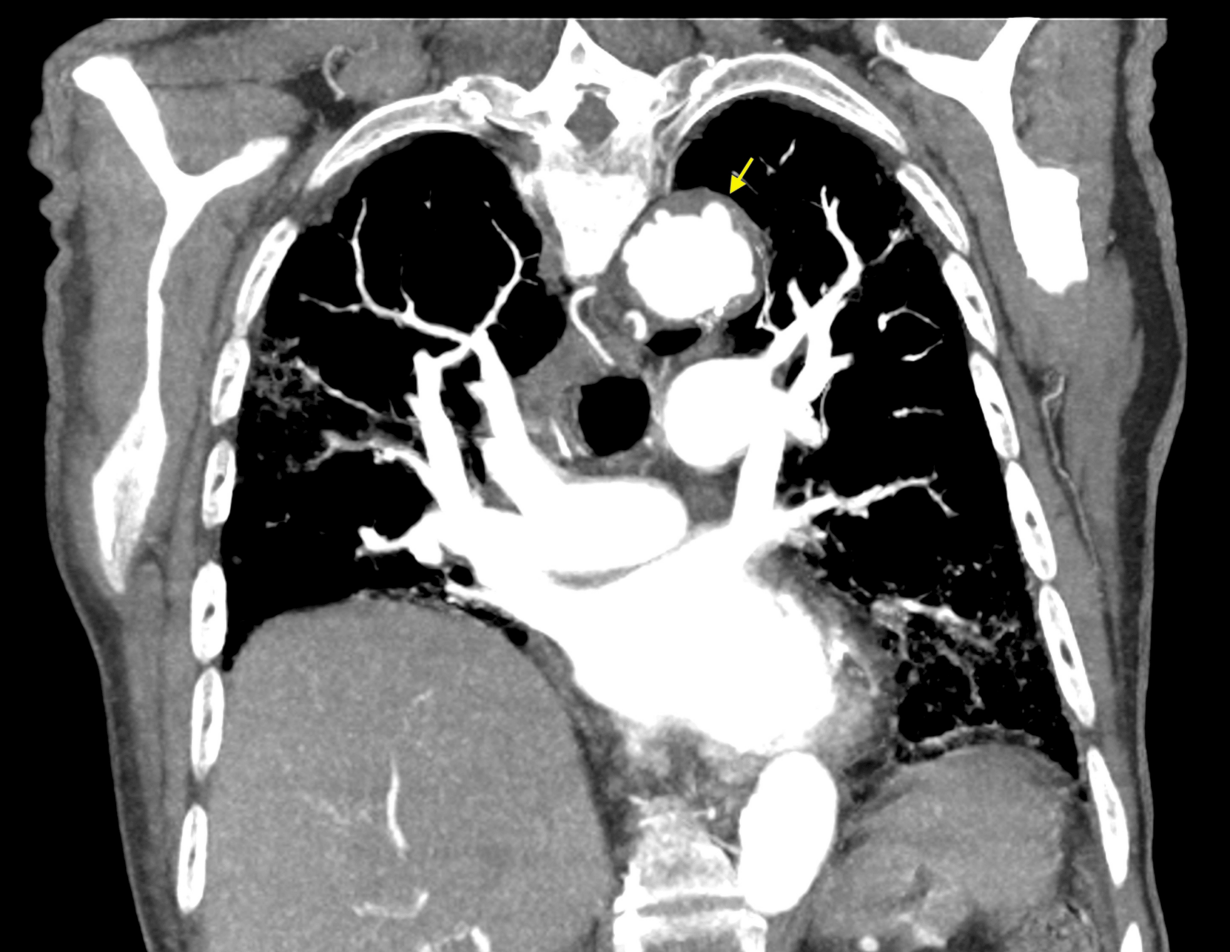
CTA of the chest with contrast (coronal view) showing multiple focal outpouchings in the distal aortic arch and proximal descending thoracic aorta (yellow arrows) CTA: computed tomography angiogram

A chest X-ray revealed patchy interstitial opacities in the bilateral lung fields, more pronounced in the right lateral upper lung and left lower lung, mildly increased compared to imaging four months prior, likely secondary to the progression of chronic parenchymal disease or a superimposed infection (Figures 1, 2). At that time cardiology and pulmonology services were consulted, and the patient was prescribed steroids and antibiotic therapy for concerns of pneumonia and exacerbation of pulmonary fibrosis. The patient had improved his respiratory symptoms, and antibiotics were discontinued due to low suspicion of pneumonia. However, due to the patient’s history of pulmonary embolism and a presentation concerning a pulmonary embolism, the patient underwent a computed tomography angiogram (CTA) with and without contrast. The CTA showed no evidence of a pulmonary embolism, mildly progressed pulmonary fibrosis compared to two years ago (Figure 3), severe coronary arterial calcification, and moderate soft and calcified atherosclerotic plaques of the aorta. In addition, numerous focal outpouchings from the distal aortic arch and proximal descending thoracic aorta were noted on the CTA with contrast (Figures 4, 5, 6).

There were at least 12 focal outpouchings found that were compatible with PAUs, which were either new or increased in size from previous imaging, becoming detectable. The most proximal PAUs were noted near the left common carotid artery, and the most distal PAUs were located near the level of the tenth thoracic vertebra, with most of the PAUs measuring up to 7 mm deep. Additionally, a ductus diverticulum was identified in the proximal descending thoracic aorta, measuring 1.1 cm in diameter and 2.5 cm in length (Figure 4). There was no evidence of hyperdensity on the non-contrast imaging studies to suggest any active hemorrhage and no evidence of dissection. Cardiothoracic surgery was consulted for the multiple PAUs, and the team recommended the patient be started on a high-dose statin (i.e., atorvastatin 80 mg daily) with strict blood pressure control using losartan 25 mg daily, with outpatient follow-up in three months for a repeat CTA to check for disease progression.

## Discussion

AAS encompasses three serious aortic conditions: acute aortic dissection, IMH, and PAU, all of which share similar characteristics. These syndromes arise from a common process that leads to the degeneration of the internal elastic lamina and media of the aorta. Stanson et al. were the first to describe PAUs of the aorta as a distinct aortic disease [1]. PAU occurs when an atherosclerotic plaque in the aorta ulcerates and penetrates the vessel's internal elastic lamina and media. Although PAUs account for only 2-7% of all AAS cases, they can result in IMH, pseudoaneurysm, or aortic rupture due to progressive aortic enlargement, with a heightened risk in the ascending aorta (Type A PAU) [1,2]. This condition typically affects older male patients with a history of smoking, hypertension, coronary artery disease, and chronic obstructive pulmonary disease, and is usually found in areas with extensive atherosclerosis, most commonly in the middle and lower descending thoracic aorta (Type B PAU) [1,3]. PAUs are infrequently found in the arch of the aorta or abdominal aorta, and rarely in the ascending aorta [1,2]. PAUs in non-contrast studies appear as focal outpouchings or irregularities in the aortic wall, often associated with localized thickening or adjacent calcifications [1,2,10]. Contrast-enhanced CT, featuring both axial and multiplanar reformations, is the preferred imaging technique for diagnosing PAU, which can resemble an IMH on non-contrast CT [1,2,10]. The key diagnostic feature is localized ulceration that penetrates the aortic intima into the aortic wall, typically appearing as an out-pouching of contrast media through a calcified plaque in the mid-to-distal third of the descending thoracic aorta [2,10]. Multiple PAUs are rarely identified simultaneously, with the rarity increasing as the number of PAUs identified grows. The simultaneous identification of seven or more PAUs in the aorta has rarely been reported [3-10].

This case adds to the limited literature discussing the incidental discovery of multiple PAUs and an associated ductus diverticulum. As discussed, this patient’s CTA with contrast revealed at least 12 focal outpouchings compatible with PAUs in the distal aortic arch and proximal descending thoracic aorta, with the most proximal near the left common carotid artery and the most distal near the 10th thoracic vertebra, measuring up to 7 mm deep (Figures 4-6). Additionally, a ductus diverticulum was found in the proximal descending thoracic aorta, measuring 1.1 cm in diameter and 2.5 cm in length (Figure 4). Management for PAU-related AAS aims to prevent aortic rupture and progression to acute aortic dissection [1-2]. Medical management with antihypertensive agents is recommended for patients with uncomplicated PAU [1,11]. Whereas endovascular or surgical intervention is recommended in complicated cases, such as recurrent pain despite medical treatment, signs of contained rupture such as rapidly growing ulcers, periaortic hematoma, or pleural effusion, and asymptomatic PAUs with a large diameter of over 20 mm or depth over 10 mm, though size criteria are debated [11,12]. This patient’s CT non-contrast images showed no active hemorrhage or dissection, and the PAU depth was less than 10 mm, but due to the presence of multiple PAUs cardiothoracic surgery was consulted, and the patient was started on high-dose statin therapy with a follow-up CTA in three months to monitor disease progression.

PAU may present similarly to aortic dissection with symptoms such as abrupt severe chest pain or mid-back pain; anterior symptoms are more common in the ascending aortic lesions while intrascapular or back pain is more common in descending aortic lesions [13-16]. Due to PAUs being typically localized lesions, they do not generally present with aortic regurgitation, pulse deficits, or visceral ischemia [2]. Symptoms more commonly occur in elderly patients, with 75% of patients with PAU experiencing pain in the mid-back or chest, but asymptomatic lesions may be identified incidentally during imaging for other suspected conditions [16,17]. PAUs begin in the intima and are usually asymptomatic initially, but as they progress into the medial layer, they can cause mid-back or chest pain [18]. As a result of PAU being a relatively rare condition and their overlapping, vague, or asymptomatic presentations, PAU diagnosis is commonly delayed until other more common conditions with similar presentations have been excluded [12,17]. This is of concern since PAUs can lead to serious life-threatening complications, such as aortic dissection, aortic rupture, and aortogastric fistula [1,2,12]. Therefore, PAU must be considered when symptoms suggest an aortic dissection or atypical chest pain to avoid missing this potentially life-threatening condition.

In this case, the patient initially presented with right-sided upper quadrant pain and pleuritic chest pain radiating to the back and tachypnea. The patient's chest X-ray showed increased patchy interstitial opacities, suspected to be due to chronic parenchymal disease progression or a superimposed infection. After treatment with steroids and antibiotics, the patient's respiratory symptoms improved. As a result, the patient was diagnosed with an acute exacerbation of pulmonary fibrosis. Given the patient's history of pulmonary embolism and tachypnea, a CTA was performed, which showed no pulmonary embolism but revealed mild pulmonary fibrosis progression, moderate aortic atherosclerotic plaques, and multiple focal outpouchings in the distal aortic arch and proximal descending thoracic aorta. However, despite PAU commonly presenting with chest pain radiating to the back, it was not considered as part of the differential diagnosis for this patient’s chest pain likely due to the patient’s additional atypical symptoms such as right-sided upper quadrant pain and improvement of respiratory symptoms with steroids. This case highlights the importance of considering PAU as part of the differential diagnoses when a patient presents with chest pain to allow early identification of this condition and prevent life-threatening complications as a result of delayed diagnosis and treatment.

## Conclusions

This case provides important insights into PAUs by documenting the incidental discovery of multiple PAUs and an associated ductus diverticulum, revealing at least 12 focal outpouchings in the distal aortic arch and proximal descending thoracic aorta. It highlights the rarity and complexity of PAU presentations and contributes to the limited data on the simultaneous identification of multiple PAUs. Furthermore, the case underscores the critical need to include PAU in the differential diagnosis for patients with chest pain, especially when symptoms are atypical or overlap with other conditions, to prevent life-threatening complications such as aortic dissection or rupture. Early recognition and appropriate management are crucial for improving patient outcomes and avoiding severe consequences associated with delayed diagnosis and treatment of PAUs.
